# Review of Tail Biting in Pigs – *a comprehensive guide to its aetiology, impact and wider significance in pig management*

**DOI:** 10.1017/awf.2025.20

**Published:** 2025-03-31

**Authors:** Birte L Nielsen

**Affiliations:** Universities Federation for Animal Welfare, United Kingdom

In the Introduction to the book, the two editors outline its content with a brief description of each of the chapters. Following three introductory chapters on legislation, anatomy and physiology, and evolutionary perspectives, the book is divided into four parts on risk factors associated with the pigs themselves and their social (3 chapters) and physical environment (3 chapters), followed by methods on addressing the problem (4 chapters), and finishing with a section on the bigger picture (3 chapters), ending with conclusions from the editors. The outline of each chapter given in the Introduction is very informative, and I will not try to emulate it here, but instead attempt to review the book as a whole.

At first glance, I was surprised to see the length of the book was 429 pages, but the word ‘*comprehensive’* in the title should have warned me. Given the length and the subject, a certain degree of overlap between some of the chapters was to be expected. However, the way each chapter is written allows all chapters to be a stand-alone read, covering a specific aspect of tail biting, but without starting from scratch each time. And with over 100 pages of references in total, this is the most comprehensive book on our current knowledge on tail biting in pigs to date.

Although I have never worked on the subject of tail biting as a researcher, anyone who has worked with growing pigs in general is aware of the issue, and that it is caused by many factors. But I had perhaps not realised just *how* complex this is – and why it is still a problem crying out for solution(s). The book is full of interesting facts, and the observation that tail biting is never observed in wild boar or feral pigs, and is a behaviour associated with domestic breeds of pigs when kept in unsuitable environments, is fundamental in guiding the search for ways to prevent and stop tail biting. The book informs the reader of the (potential) evolutionary background to this non-adaptive behaviour (Chapter 3) and why tail docking has been the main remedy to prevent tail biting. The chapter on the anatomy of the pig tail by Sandercock, Herskin, and Nordgren (Chapter 2) was particularly eye-opening, as it drove home the severity of cutting off a tail; a procedure seen by some as relatively harmless. I also learned that the curled tail is a feature of domestic pigs only, as wild boars do not curl their tail. It was made clear that tail docking, just like a bitten tail, can cause long term pain, due to the high level of somatosensory innervation of the pig tail. The book also teaches us about the functionality of the tail in pigs, including protecting the hind-area of the animal, swiping away insects, and as a reflection of the emotional state of the pig.

Taking into account the functionality of an intact tail and the persistent pain caused by cutting or biting it, it is shocking that tail docking, despite being outlawed as a routine practice in the European Union, is still carried out on around 150 million pigs annually in the EU (Chapter 1). Given the extent of the problem and the methods currently used – more or less successfully – to prevent it, why have we not found a solution already? Because it is super complex. Having read this book, I am left with the impression that the causes of tail biting is the mothership of ‘multi-factorial’. The list includes, in no particular order, sex; breed; birth weight; growth rate; health problems; immune system (Chapter 5); cross-fostering; noticeable changes in the environment (but not always); group size and stocking density; group activity, dynamics, and synchronisation (Chapter 6); pen structures and layout; floor type; temperature and humidity (Chapter 7). I will let you catch your breath before continuing.

Other factors are the presence, quality, type, freshness, mode of presentation and quantity of rooting material and other environmental enrichment (Chapter 8). Enrichment is thought to reduce tail biting by alleviating boredom, satisfying an innate foraging motivation, and as an outlet for stress. Adding to the list of causal factors is the amount, quality, composition, form, accessibility and pattern of feed supply (Chapter 9, where one piece of concrete advice is to sprinkle salt on the pen floor in case of a tail-biting outbreak); emotional contagion; draught; air quality; light intensity and noise. Shall I continue?

Some of these factors affect the risk of tail biting indirectly, such as when high humidity leads to a decrease in the amount of lying, which in turn may contribute to more tail biting. Many of the factors mentioned have been found to increase *and* decrease the risk of tail biting, e.g. noise may act as a stressor (increase) or as a distraction (decrease). Tail biting can spread slowly or quickly through a group (Chapter 6), only certain pigs bite (Chapter 4), and the victims of tail biting are not random (Chapter 3). Because of this erratic nature of tail-biting outbreaks, it is difficult to study and to measure consistently (Chapter 11), which is a “*contributing factor to inconclusive or inconsistent results across studies*” (Chapter 4).

I was surprised to discover that it is difficult to ascertain if a docked tail has been bitten and subsequently healed (Chapter 11), but that a fully intact tail can be clearly identified by its flattened last vertebrae (Chapters 2 and 11). Part of the difficulty in the study of tail biting is not knowing how long the tail was supposed to be, i.e. what proportion of the tail is missing? Based on only a small sample, the tail of the domestic pig has been estimated at just under 10 cm at birth, growing to just over 30 cm at 5 months of age (Herskin *et al.* 2015).

The chapter on risk assessment by Dippel (Chapter 10) gives a useful overview of many of these factors, and states that – once producers have adapted to not docking the tails of their pigs – similar levels of tail biting can be achieved in docked and non-docked pigs. The author recommends frequent inspection (more than once a day) of all pens, checking if all pigs look good, do not behave differently from usual, and have all the necessary provisions.

In the chapter on ethical aspects of tail biting and docking (Chapter 16), Bovenkerk, Bracke, and Valros raise the notion that if docking is deemed necessary, then the environment in which the pigs are kept is not suitable. This links nicely to the notion of using intact, curly tails as an iceberg indicator (Chapter 13) – a single indicator that captures a number of welfare issues. The tail lends itself well to this as it is readily observable at all times.

The financial costs of tail-biting lesions (additional work, medication, reduced productivity) across Europe are estimated, according to Chapter 15, to be around €0.6 to €3.4 per finished pig. If only we could monitor various indicators to automatic detect or even predict tail-biting behaviour (Chapter 13), but results so far are not promising (yes, you guessed it: it’s multifactorial), and limited by current sensors measuring variables that are not specific to tail damage. One of the oft-cited references in the book is the 2007 EFSA Opinion on tail biting and its possible causes, where the main factors are listed as absence of straw, presence of slatted floors, and a barren environment. This gives me an excuse to highlight a particularly interesting section in Chapter 15 by Niemi and Stevenson. They cite the wording from the EU Pig Directive (European Council 2008), where it says that ‘*pigs must have permanent access to a sufficient quantity of material to enable proper investigation and manipulation activities, such as straw, hay, wood, sawdust, mushroom compost, peat or a mixture of such, which does not compromise the health of the animals*’, and go on to remind us that the use of ‘*such as’* in legal language means something of a similar nature to the examples given – i.e. natural, destructible, chewable, manipulable, and ingestible substrates, and not metal chains or rubber dog toys.

Overall, this is an excellent book that covers a lot of ground on a subject that continues to baffle both scientists and pig producers. The book is expensive to buy compared to similar types of textbooks, but some of the chapters (7 out of 16) are available as open access. Some of the chapters may be less accessible for the lay-person or non-scientist but, as the ultimate work on the subject of tail biting in pigs, this book is a must-read for anyone who has an interest in the welfare of growing pigs.
